# Environmental and Organizational Factors Associated with Elbow/Forearm and Hand/Wrist Disorder among Sewing Machine Operators of Garment Industry in Ethiopia

**DOI:** 10.1155/2014/732731

**Published:** 2014-09-15

**Authors:** Wakjira Kebede Deyyas, Ararso Tafese

**Affiliations:** ^1^Department of Medical Laboratory Sciences and Pathology, Collage of Public Health and Medical Sciences, Jimma University, P.O. Box 378, Jimma, Ethiopia; ^2^Oromia Regional Social and Labour Affairs Agency, Addis Ababa, Ethiopia

## Abstract

Occupational health problems related to upper limp musculoskeletal disorders were the major issue among sewing machine operators of garment industries in Ethiopia. The aim of this study was to assess the prevalence and associated risk factors of work related elbow and wrist musculoskeletal disorders among sewing machine operators of garment industries in Galan City, Oromia Regional State. A cross-sectional study was conducted from April 1 to 30, 2012. A total of 422 study subjects were included in this study. Standard Nordic Musculoskeletal Questionnaire was used to collect detailed information on musculoskeletal symptom, sociodemographic data, and factors associated with the problems through face to face interview. From a total of 422 sewing machine operators included in the study 370 (87.7%) were females and 306 (72.5%) were in the age group of <30 years. The prevalence of self-reported work related elbow and wrist musculoskeletal disorders was 40% and 37.7%, respectively. In multivariate analysis, those who had >16 years of service were about five times more likely to develop elbow and wrist musculoskeletal disorders than those who had short (1–5 years) year of services [AOR = 4.7, 95% CI: 1.55–13.02], physical activities [AOR = 5.02, 95% CI: 1.57–16.00], and methods of payment [AOR = 2.01, 95% CI: 1.23–3.28], factors significantly associated with this disorders. Work related elbow and wrist musculoskeletal disorders were high among sewing machine operators in selected garment industries. Moreover, personal and environmental factors were identified as the potential risk factors related to elbow and wrist musculoskeletal disorders among the study group. Therefore, government and the owner of the garment industries should give special attention to prevent and control the problems through proper occupational health and safety policy implementation in the country.

## 1. Introduction

Work related musculoskeletal disorders (WRMSDs) have been considered as the potential threats of major public health problems associated with unsafe work environments. The daily hardship cost of human is the burden of poor occupational safety and health practices that can cause pain and disability in the functions of upper body musculoskeletal systems. About 6,300 individuals were dying every day as a result of occupational work related problems in the world [1]. The issues of safety and health conditions at workplace were different between countries, organizational sectors, and social groups. For example, about 317 million accidents occur on the job annually, mostly which take a heavy toll in developing countries where thousands of poor and the least protected people were involved in sewing machine operator in garment industries [2].

WRMSDs among sewing machine operators of garment industries were a reason for long term sick leave and disability pensions in the world [3]. World Health Organization (WHO) reported 50–70% individuals developed WRMSDs when exposed to poor working environments during working for the job [4]. A study conducted in Boston, America, showed that sewing machine operators had been highly affected by work related upper limb pain. Moreover, the United State Bureau of Labour Statistics Annual Survey of occupational injuries and illnesses ranked sewing machine operators of clothing industry in the country offering to upper body musculoskeletal disorders [5].

Study in Finland among office employees reported that female workers were more likely exposed to WRMSDs than male workers [5]. Moreover, Turkey and Denmark reported the years of service were significantly associated with elbow, wrist, and shoulder and neck musculoskeletal (MS) disorders among workers in garment industries [6–8]. In Denmark population the prevalence of MS symptoms of the neck, shoulders, back, and wrist among sewing machine operators were found to be 57%, 51%, 47%, and 26%, respectively [7]. Moreover, results from 11 factories from 7 sectors of manufacturing industry in South Africa exposed to poor design of working environments in the workplace were significantly associated with MS pain of the hand, elbow, neck, and shoulders [9].

Harmful inflammatory diseases that can cause pain and disability in the functions of elbows, arms, wrists, and hands experienced in the last 12 months were used as an outcome measure for this study. Therefore, prevention of WRMSDs among workforces may be a national priority in many countries. Even though an Ethiopian labour proclamation permits working in all types of industries for 8 hours per day or 48 hours per week [10], there is a lack of strong functioning health and safety system, particularly, among garment industries where workers are involved in sewing machine operating tasks in the study area.

This study aimed to assess the prevalence and associated risk factors of work related elbow/forearm and wrist/hands MS disorders among workers who were directly involved in operating sewing machine work in the selected garment industries in Galan City. The findings of this study will provide information on workplace illness and injuries in the garment industries and also have important public health implication by providing information for policy makers to design strategy to keep strong functioning health and safety system for people who become tackled by these problems.

## 2. Materials and Methods

### 2.1. Study Setting and Period

An institution based cross-sectional study was conducted in two large garment industries selected in Galan City administration from April 1 to 30, 2012. Galan City is one of the industrial zones in Oromia Regional State, which is 25 KMs far from Addis Ababa, the capital city of Ethiopia, in Eastern direction, where most of the foreign and domestic investors were investing in different industrial activities. Among these industrial activities, operating garment factories were the most common ones. Two large garment industries, owned by Ethiopians investors living outside Ethiopia, were selected for this study, each having 632 and 362 sewing machine operators, respectively.

### 2.2. Sample Size and Sampling Procedures

A total of 994 sewing machine operators who had worked in the two garment industries for more than 12 months prior to the study period were considered as source population. The sample size was determined using single population proportion formula and, to maximize the sample size, 50% assumption was assumed at 95% confidence interval and margin of error 5%. Therefore including 10% nonresponse rate, the total sample size obtained was 422. First, sewing machine operators were stratified by its organizational structure and then afterwards the sample size was proportionally allocated to each garment industry. Lottery method was used to recruit the actual number of study units. Administrative or supportive staff and workers who were absent during data collection period were excluded from the study.

### 2.3. Data Collection Process

Data were collected using a standardized Nordic questionnaire [11]. The questionnaire was translated from English into Amharic and then independently back-translated to English with adjustment of the Amharic version where problems were identified. The contents of the questionnaire included sociodemographic characteristics (sex, age, marital status, educational level, monthly salary, and years of service), personal factors (height and weight of participants were measured to calculate BMI, smoking behavior, habit of doing physical activity, medical history of systemic illness, and medical history of musculoskeletal disorder), organizational factors (payment method, work hours, employment status, break time excluding lunch, and health and safety training), and working environmental factors (job satisfaction, repetitive work within less than 30 seconds, doing too much work, availability of sufficient light, and availability of adjustable chair). At baseline, participants were asked about pains in the elbow/forearm and wrist/hand lasting for at least a day over the past 12 months and those who had reported pain at baseline over the past 12 months were determined.

### 2.4. Ethical Clearance

Ethical clearance was obtained from Oromia Regional Labour and Social Affairs Agency. A formal letter of cooperation was delivered to the owner of the garment industries. Verbal informed consent was obtained from each study participant after having been informed of the objective of the study.

### 2.5. Data Analysis and Interpretation

The collected data were edited, coded, and entered into SPSS version 20.0 software program for analysis. Bivariate logistic regression analysis was used to determine the effect of independent variables on the outcome variables and those variables with *P* value < 0.2 were exported to multiple logistic regression for further analysis [12], and the summary statistics such as mean, standard deviation, and percentage were used to describe the study population in relation to outcome variables. The degree of association between independent and dependent variables was assessed using odds ratio with 95% confidence interval.

## 3. Results

### 3.1. Sociodemographic Characteristics of Study Participants

A total of 422 sewing machine operators were included in the study and are with 100% response rate. Majority of the study subjects, 370 (87.7%), were females. Most of the respondents, 306 (72.5%), were in the age group of <30 years and the mean age of the study subjects was 26.9 ± 7.2 SD. For marital status, 227 (53.8%) were single and 145 (34.4%) were married and 200 (47.4%) had attended secondary school. Among the respondents, 270 (64.0%) had served from 1–5 years and 23 (5.3%) had served above 16 years and the mean years of service were 5 (with SD 5.27, minimum and maximum 1 and 30, resp.). Two hundred and fifty-two (59.7%) were paid monthly salary of 700 to 900 Ethiopian birr or 35.5 to 45.5 US dollar and 138 (32.7%) were paid below 700 Ethiopian birr or below 35.5 US dollar per month (Table 1).

### 3.2. Personal Characteristics of Study Participants

Body mass index of the respondents was calculated by measuring height and weight of the participants during data collection by trained nurses and 325 (77.0%) subjects were being healthy (18.6–24.9 kg/m^2^) and 4 (0.9%) were obese (>30 kg/m^2^) and the mean body mass index of the study subjects was 20.4 ± 2.62 SD with minimum and maximum value of 13.5 and 31.25, respectively. Three hundred and fifty-four (83.9%) were not practicing physical exercise, 26 (6.2%) were practicing more than three times per week. Three hundred and ninety-five (93.6%) of the respondents were noncigarette smokers, 65 (15.4%) were with medical history of MS disorders, and 43 (10.2%) were with systemic illness (Table 2).

### 3.3. Conditions of Working Environment of Sewing Machine Operators

Almost half of the respondents, 204 (48.3%), were not satisfied with their current job, 196 (46.4%) of the respondents were always exposed to repetitive task with repeating the same task within 30 seconds, and 200 (47.4%) of the respondents feel always doing too much work within working hour. Three hundred and seventy-one (87.9%) of the respondents were having enough sufficient light to operate sewing machine, and 307 (72.7%) of the operators were not using an adjustable chair (Table 3).

### 3.4. Organizational Factors of Sewing Machine Operators

Employment status of respondents, 397 (94.1%), was permanent and the rest were temporary workers. Three hundred and thirty (74.2%) of the operators' payment method was by means of hourly rate. Among 380 (90.0%) of the workers who worked for 8 hours per day, 343 (81.3%) of the workers were not having break (excluding lunch time). The majority of 405 (96.0%) of the respondents had never attained training on the issue of ergonomic at workplaces (Table 4).

### 3.5. Prevalence of Elbow/Forearm and Hands/Wrist MS Disorders

The prevalence of work related elbow/forearm and hands/wrist MS disorders among sewing machine operators who had experienced trouble (ache, pain, and discomfort) in the last 12-month period were 40% and 37.7%, respectively. One hundred and sixty-nine (40.0%) of the respondents had elbow/forearm MS disorders. Of  these, 70 (16.6%) had experienced ache, pain, and discomfort more than 30 days, but not every day. Of the total respondents, 159 (37.7%) of the sewing machine operators developed hand/wrist MS disorders, of which 50 (11.8%) had experienced pain and discomfort for more than 30 days (Table 5).

### 3.6. Factors Associated with Work Related Elbow/Forearm and Hand/Wrist MS Disorders

In bivariate logistic analysis, variables such as gender, educational level, monthly salary, year of service, habit of doing physical activities, employment status, payment method, and medical history of systemic illness were significantly associated with elbow/forearm MS disorders; however, after adjusting for the effects of other variables gender and monthly salary were independently associated with each other. Moreover, year of service, habit of doing physical activities, and payment methods were significantly associated with elbow/forearm disorders. Being males to be a risk for the problems than females [AOR = 1.8, 95% CI: 1.7–4.8]. Those who had service of >16 years were about 5 times more likely to develop elbow/forearm disorder than those who had short (1–5 years) years of service [AOR = 4.7, 95% CI: 1.55–13.02].

Workers who did not have habit of doing physical activities were 5 times more likely to develop elbow/forearm disorder than those who had the habit of doing physical activities more than three times per week [AOR = 5.02, 95% CI: 1.57–16.00] and workers whose payment was via piece rate were 2 times more likely to develop elbow/forearm disorder than those paid through hourly payment [AOR = 2.01, 95% CI: 1.23–3.28] (Table 6). Employees with longer years of service (≥16 years) were 4 times more likely to develop hand/wrist disorder than those who had short (1–5 years) years of service [AOR = 4.09, 95% CI: 1.48–11.24] (Table 7).

## 4. Discussion

Work related MS disorders were common problems in garment industries, especially among sewing machine operators in the study area. In the present study, the prevalence of self-reported elbow/forearm and hand/wrist MS disorders was 40% and 37.7%, respectively. This finding was comparable with the studies done in UK and Finland where the prevalence of hand MS disorders was reported to be from 34 to 75%, [13, 14]. However, a study in Los Angeles, USA, showed that the prevalence of elbow hand/wrist was  16% [15]. This difference might be due to the difference of the study setting. In the USA the study was conducted on small shop sizes in which participants could mostly be the family or relatives of the shop owners and therefore they were not enforced to work for long time. However, this study was conducted on large scale industries in which workers were working for more than eight hours per day.

In this study, males sewing machine operators were more likely to develop elbow/forearm and hand/wrist disorders than females sewing machine operators [AOR = 1.8, 95% CI: 1.7–4.8] and [AOR = 2.9, 95% CI: 1.3–6.4], respectively. Studies conducted among Iranian sewing machine operators of shoe manufacturing showed that females were more at risk to develop elbow MS disorders [7, 16]. This difference might be due to the deference between the type of machines used in shoe manufacturing and garment industries. Additionally, males were interested to do work even during night time.

The increment of wages of employees in this study setting was depending on their year of service in the same industry. The chance of having to develop work related MS disorders could be higher among highly paid employees than those who were paid less. Employees with longer years of service (≥16 years) in garment industry were about 4 times more likely to develop elbow/forearm and hand/wrist disorder than employees who had short (1–5 years) years of service, [AOR = 4.70, 95% CI: 1.55–13.02] and [AOR = 4.09, 95% CI: 1.48–11.24], respectively. This reveals that operating sewing machine for longer years was strongly associated with increased work related elbow and hand/wrist MS disorders. This finding was in agreement with the study conducted in the Los Angeles, Turkey, Iran, and Denmark [5, 7, 11, 17].

Habits of doing physical exercise had significant association with work related elbow/forearm and hand/wrist MS disorders. Employees who had no habits of doing physical exercises were about 3 up to 5 times more likely to develop hand/wrist and elbow/forearm disorder than workers doing physical exercise for more than three times per week [AOR = 3.41, 95% CI: 1.11–10.44] and [AOR = 5.02, 95% CI: 1.57–16.05], respectively. However, those practicing once per week were more likely to develop elbow/forearm and hand/wrist disorder than those practicing more than three times per week. This finding was comparable with a study conducted among workers of video display unit in Finland [18].

In contractual agreement of employment, temporary workers (definite period contract agreement) were 3.39 times more likely to develop hand/wrist MS disorders than permanent (indefinite period) workers. However, the Ethiopian labour proclamation number 377/2003, under article 9, 10 stated that “Any contract of employment should be deemed to have been concluded for an indefinite period except for working activates require definite period contract agreement.” This finding has also exceeded the standard set in Ethiopian labour proclamation number 377/2003 which states that* “The time during which a worker actually performs work or avails herself/himself for work in accordance with law, collective agreement or work rules shall not exceed eight hour a day or forty-eight hour a week”* [10].

The payment method affects the prevalence of elbow/forearm and hand/wrist MS disorders among sewing machine operators of garment industries in the study area. Employees with payment method by piece rate were about 2 times more likely to develop elbow/forearm and hand/wrist disorders than those who were paid by hourly rate [AOR = 2.01, 95% CI: 1.23–3.28] and [AOR = 1.78, 95% CI: 1.08–2.93], respectively. This might be due to the reason that workers increase their speed of work to get more payment. This finding was in agreement with the study conducted in Los Angeles, USA, among sewing machine operators [15].

Generally, there is high prevalence of elbow/forearm and hand/wrist MS disorders among sewing machine operators in the study area. The independent variables such as monthly salary, years of service, physical activities, employment status, and methods of payment had statistically significant association with the occurrence of work related elbow/forearm and hand/wrist MS disorders. Promoting worker involvement in efforts to improve workplace conditions is a critical element in organization process to enhance worker motivation/job satisfaction, added problem solving capabilities, and greater knowledge of work experiences.

Moreover, training is an essential element for any effective safety and health program to recognize workplace risk factors associated with MS disorders and to understand general methods for controlling them and identify the signs and symptoms of MS disorders that may result from exposure to organizational and environmental factors. Furthermore, this study suggested that previous medical history of MS disorders and systemic illness, piece rate work, and a length of working hours per day were key issues which require specific interventions to deal with. Therefore, concerned body gives special attention to prevent and control work related elbow/forearm and hand/wrist MS disorders in garment industries through proper occupational health and safety policy implementation.

## Figures and Tables

**Table 1:** Sociodemographic characteristics of study participants and year of service in garment industries in Galan City, Oromia Regional State, Eastern Ethiopia, 2012.

Category of variable	Frequency (*n* = 422)	(%)
Sex		
Male	52	(12.3)
Female	370	(87.7)
Age (in years)		
>30 years	306	(72.5)
30–39 years	89	(21.1)
40–49 years	18	(4.30)
≥50 years	9	(2.10)
Marital status		
Married	145	(34.0)
Divorced	29	(6.80)
Widowed	21	(5.00)
Single	227	(58.8)
Educational level		
Illiterate	45	(10.7)
Primary	135	(32.0)
Secondary	200	(47.40
Higher education	42	(10.0)
Monthly salary		
<700 ETB (<35.5 US dollar)	138	(32.7)
700–900 ETB (35.5–45.5 US dollar)	252	(59.7)
>900 ETB (>45.5 US dollar)	32	(7.60)
Year of service in garment		
Mean (±SD)	5 (±5.2)	—
1–5 years	270	(64.0)
6–10 years	118	(28.0)
11–15 years	11	(2.60)
≥16 years	23	(5.40)

**Table 2:** Personal factors associated with elbow and wrist MS disorder among sewing machine operators in garment industries in Galan City, Oromia Regional State, Eastern Ethiopia, 2012.

Category of variable	Frequency (*n* = 422)	(%)
BMI (body mass index)		
Underweight (<18.5 kg/m^2^)	76	(18.0)
Healthy (18.5–24.9 kg/m^2^)	325	(77.0)
Overweight (25–29.9 kg/m^2^)	17	(4.1)
Obese (≥30 kg/m^2^)	4	(0.9)
Habit of doing physical activities		
None	354	(83.9)
Once per week	29	(6.9)
Two times per week	13	(3.1)
≥Three times per week	26	(6.2)
Smoking behaviour		
Nonsmoker	395	(93.6)
Past smoker	14	(3.3)
Current smoker	13	(3.1)
Medical history of MS disorders		
Yes	65	(15.4)
No	357	(84.6)
Medical history of systemic illness		
Yes	43	(10.2)
No	379	(89.8)

**Table 3:** Working environmental factors associated with elbow and wrist MS disorders among sewing machine operators in garment industries in Galan City, Oromia Regional State, 2012.

Category of variables	Frequency (*n* = 422)	(%)
Job satisfaction Not satisfied Somewhat satisfied Very satisfied Repetitive work within < 30 seconds Never Sometimes Often Always Doing too much work Never Sometimes Often Always Availability of sufficient light Yes No Adjustable chair Yes No	204 187 31 13 96 117 196 8 123 91 200 371 51 115 307	(48.3) (44.3) (7.4) (3.1) (22.7) (27.7) (46.5) (1.9) (29.1) (21.6) (47.4) (87.9) (12.1) (27.3) (72.7)

**Table 4:** Organizational factors associated with elbow and wrist MS disorders among sewing machine operators in garment industries in Galan City, Oromia Regional State, Eastern Ethiopia, 2012.

Category of variables	Frequency (*n* = 422)	(%)
Employment status Temporary Permanent Payment method Hourly payment Piece rate payment Total working hours a day 8 hours 9-10 hours >10 hours Total working breaks excluding lunch break ≤15 minutes >15 minutes None Training on issue of ergonomics Yes No	25 397 313 109 380 25 17 42 37 343 17 405	(5.9) (94.1) (74.2) (25.8) (90.0) (5.9) (4.1) (10) (8.8) (81.2) (4.0) (96.0)

**Table 5:** Prevalence of elbow/forearm and hands/wrist MS disorders among sewing machine operators in the last 12 months in garment industries in Galan City, Oromia Regional State, 2012.

	Symptoms by anatomical body site	
	Elbow/forearm disorder, *N* (%)	Hand/wrist disorder, *N* (%)
Trouble in the past 12 months		
Yes	169 (40.0)	159 (37.7)
No	253 (60.0)	263 (62.3)
Total length of MS disorder trouble		
1–7 days	34 (8.1)	37 (8.8)
8–30 days	40 (9.4)	36 (8.5)
>30 days, but not every day	70 (16.6)	50 (11.8)
Every day	25 (5.9)	36 (8.5)

**Table 6:** Multivariate analysis for factors associated with elbow/forearm MS disorders among sewing machine operators in Galan City, Oromia Regional State, Eastern Ethiopia, 2012.

Category of variables	Elbow/forearm disorder			
	Yes *n* (%)	No *n* (%)	COR (95% CI)	AOR (95% CI)
Sex				
Male	34 (65.4)	18 (34.6)	3.2 (1.6–5.5)	1.8 (1.7–4.8)∗∗
Female	135 (36.5)	235 (63.5)	1.00	1.00
Educational level				
Illiterate	17 (37.8)	28 (62.2)	0.55 (0.23–1.29)	
Primary school	59 (43.7)	76 (56.3)	0.70 (0.35–1.41)	
Secondary school	71 (35.5)	129 (64.5)	0.50 (0.25–0.97)	
Higher education	22 (52.4)	20 (47.6)	1.00	
Monthly salary				
<700 ETB (<35.5 US dollar)	34 (24.6)	104 (75.4)	0.17 (0.07–0.39)	0.21 (0.08–0.54)∗
700–900 ETB (35.5–45.5 US dollar)	114 (45.2)	138 (54.8)	0.43 (0.20–0.93)	0.47 (0.19–1.13)
>900 ETB (>45.5 US dollar)	21 (65.6)	11 (34.4)	1.00	1.00
Year of service in garment				
1–5 years	93 (34.5)	177 (65.5)	1.00	1.00
6–10 years	52 (44.1)	66 (55.9)	1.50 (0.96–2.32)	1.04 (0.64–1.70)
11–15 years	6 (54.5)	5 (45.4)	2.28 (0.67–7.68)	1.50 (0.39–5.77)
≥16 years	18 (78.3)	5 (21.7)	6.85 (2.46–19.04)	4.70 (1.55–13.02)∗∗
Physical activities				
None	141 (39.8)	213 (60.1)	1.48 (0.63–3.51)	5.02 (1.57–16.05)∗
Once per week	17 (58.6)	12 (41.4)	3.18 (1.04–9.70)	5.54 (1.46–21.00)∗
Two times per week	3 (23.0)	10 (76.9)	0.67 (0.14–3.13)	0.59 (0.10–3.49)
≥3 times per week	8 (30.8)	18 (69.2)	1.00	1.00
Medical history of systemic illness				
Yes	30 (46.2)	35 (53.8)	2.03 (1.07–3.85)	
No	139 (38.9)	218 (61.1)	1.00	
Employment status				
Temporary	16 (64.0)	9 (36.0)	2.83 (1.22–6.37)	
Permanent	153 (38.5)	244 (61.5)	1.00	
Payment method				
Hourly payment	111 (35.5)	202 (64.5)	1.00	1.00
Piece rate payment	58 (53.2)	51 (46.8)	2.07 (1.33–3.21)	2.01 (1.23–3.28)∗
Training on issue of ergonomics				
Yes	11 (64.7)	6 (35.3)	1.00	
No	158 (39.1)	247 (60.9)	2.86 (1.04–7.90)	

AOR: adjusted odds ratio, COR: crude odd ratio, ETB: Ethiopian Birr, US: United States, *P value < 0.05, **P value < 0.001.

**Table 7:** Multivariate analysis for factors associated with hands/wrist MS disorders among sewing machine operators in Galan City, Oromia Regional State, Eastern Ethiopia, 2012.

Category of variables	Hand/wrist disorder			
	Yes *n* (%)	No *n* (%)	COR (95% CI)	AOR (95% CI)
Sex				
Male	31 (59.6)	21 (40.4)	2.79 (1.9–6.4)	2.9 (1.3–6.4)∗
Female	128 (34.6)	242 (65.4)	1.00	1.00
Educational level				
Illiterate	18 (40.0)	27 (60.0)	0.60 (0.25–1.41)	
Primary school	56 (41.5)	79 (58.5)	0.64 (0.32–1.29)	
Secondary school	63 (31.5)	137 (68.5)	0.41 (0.21–0.82)	
Higher education	22 (52.4)	20 (47.6)	1.00	
Monthly salary				
<700 ETB (<35.5 US dollar)	35 (25.4)	103 (74.6)	0.26 (0.11–0.58)	0.34 (0.14–0.85)∗
700–900 ETB (35.5–45.5US dollar)	106 (42.1)	146 (57.9)	0.56 (0.26–1.18)	0.66 (0.28–1.52)
>900 ETB (>45.5 US dollar)	18 (56.3)	14 (43.7)	1.00	1.00
Year of service in garment				
1–5 years	86 (31.8)	184 (68.1)	1.00	1.00
6–10 years	50 (42.4)	68 (57.6)	1.57 (1.00–2.45)	1.14 (0.70–1.86)
11–15 years	6 (54.5)	5 (45.5)	2.56 (0.76–8.64)	1.83 (0.47–7.03)
≥16 years	17 (73.9)	6 (26.1)	6.06 (2.30–15.91)	4.09 (1.48–11.24)∗
Physical activities				
None	130 (36.7)	224 (63.3)	1.57 (0.64–3.84)	3.41 (1.11–10.44)∗
Once per week	18 (62.1)	11 (37.9)	4.44 (1.41–13–97)	5.34 (1.44–19.76)∗
Two times per week	4 (30.8)	9 (69.2)	1.20 (0.28–5.20)	1.26 (0.24–6.41)
≥3 times per week	7 (26.9)	19 (73.1)	1.00	1.00
Smoking behaviour				
None	139 (34.9)	256 (64.8)	1.00	
Past smoker	10 (71.4)	4 (28.6)	4.60 (1.41–14.95)	
Current smoker	10 (76.90	3 (23.1)	6.13 (1.66–22.67)	
Employment status				
Temporary	18 (72.0)	7 (28.0)	4.66 (1.90–11.44)	3.39 (1.24–9.25)∗
Permanent	141 (35.5)	256 (64.5)	1.00	1.00
Payment method				
Hourly payment	102 (32.6)	211 (67.4)	1.00	1.00
Piece rate payment	57 (52.3)	52 (47.7)	2.26 (1.45–3.53)	1.78 (1.08–2.93)∗
Training on health and safety				
Yes	11 (64.7)	6 (35.3)	1.00	
No	148 (36.5)	257 (63.5)	3.18 (1.15–8.78)	

AOR: adjusted odds ratio, COR: crude odd ratio, ETB: Ethiopian Birr, US: United States, *P value < 0.05.
